# The complex roles of IL-36 and IL-38 in cancer: friends or foes?

**DOI:** 10.1038/s41388-025-03293-4

**Published:** 2025-03-08

**Authors:** Méabh Finucane, Elizabeth Brint, Aileen Houston

**Affiliations:** 1 Department of Medicine, School of Medicine, University College Cork, Cork, Ireland; 2 Department of Pathology, School of Medicine, Cork University Hospital, University College Cork, Cork, Ireland; 3 APC Microbiome Ireland, University College Cork, Cork, Ireland

**Keywords:** Tumour immunology, Cancer microenvironment

## Abstract

The interleukin-36 (IL-36) family comprises of three pro-inflammatory receptor agonists (IL-36α, IL-36β and IL-36γ), two anti-inflammatory receptor antagonists (IL-36RA and IL-38) along with the IL-36 receptor (IL-36R). Part of the IL-1 cytokine superfamily, the IL-36 family was discovered in the early 2000s due to the homology of its member sequences to the IL-1 cytokines. As pro- and anti-inflammatory cytokines, respectively, IL-36α, IL-36β, IL-36γ and IL-38 aid in maintaining homoeostasis by reciprocally regulating the body’s response to damage and disease through IL-36R-associated signalling. With the significant roles of IL-36α, IL-36β and IL-36γ in regulating the immune response realised, interest has grown in investigating their roles in cancer. While initial studies indicated solely tumour-suppressing roles, more recent work has identified tumour-promoting roles in cancer, suggesting a more complex dual functionality of the IL-36 cytokines. The activity of IL-38 in cancer is similarly complex, with the receptor antagonist displaying distinct tumour-suppressive roles, particularly in colorectal cancer (CRC), in addition to broad tumour-promoting roles in various other malignancies. This review provides a comprehensive overview of the IL-36 and IL-38 cytokines, their activation and IL-36R signalling, the physiological functions of these cytokines, and their activity in cancer.

## Introduction

The interleukin (IL) -36 cytokine family was discovered in the early 2000s due to the homology of its member sequences to the IL-1 superfamily. The IL-36 family consists of three pro-inflammatory cytokines (IL-36α, IL-36β, and IL-36γ), two receptor antagonists (IL-38 and IL-36Ra) and the IL-36 receptor (IL-36R). Since the recognition of tumour-promoting inflammation as a hallmark of cancer in 2011, there has been significant interest in the roles of the IL-36 family members in cancer, with several studies reporting anti-tumorigenic and, more recently, pro-tumorigenic functions in cancer. This review summarises current knowledge on the biology of the IL-36 and IL-38 cytokines, their production and activation. The physiological functions of these cytokines are discussed, including their roles in maintaining host homoeostasis, regulating the inflammatory immune response and protecting against bacterial and viral infection. Finally, the dichotomous anti- and pro-tumorigenic roles of IL-36 and IL-38 cytokines in cancer will be reviewed.

## IL-36 and IL-38 cytokines

The IL-36 family are classified as members of the IL-1 superfamily due to the conservation of the amino acid sequence and gene structure [1]. IL-36 encoding genes are contained in a 430 kb IL-1 gene cluster located on chromosome 2q13 between the IL-1β and IL-1RA genes [2], and possess between 20% and 50% amino acid sequence homology with IL-1 family members [3]. All six family members have also been identified in mice, with cloning experiments revealing approximately 54–91% sequence homology with human IL-36 genes. In addition, the functional roles of IL-36 are indicated to be conserved between species due to similar genomic location [1]. IL-38, in turn, is also localized within the IL-1 gene cluster, downstream of IL36RN and upstream of IL1RN, with which it shares 43% and 41% amino acid identity, respectively [4].

IL-36α, IL-36β and IL-36γ are produced by epithelial cells, keratinocytes, and a variety of immune cells, including dendritic cells (DCs), macrophages and T cells [5] and function by binding to the IL-36R, eliciting broad pro-inflammatory responses. The IL-36R is a trans-membrane receptor expressed on various epithelial cells (lung, intestine, and brain) and endothelial cells [6] along with several cells of the immune system including DCs, M2 macrophages and CD4+ T cells [7]. As regulators of the inflammatory immune response, the IL-36 family also has two anti-inflammatory members. IL-36RA is produced in embryonic and epithelial tissue (brain, stomach, and skin) [8] along with various immune cells including DCs, B cells, macrophages, and monocytes [3]. IL-36RA competitively binds to the IL-36R, preventing the binding of IL-36 agonists and subsequent pro-inflammatory gene transcription.

IL-38 is produced in various tissues, including heart, tonsil, skin, thymus, and placenta, particularly by keratinocytes and proliferating B cells [9–11]. In contrast to the IL-36RA, IL-38 not only blocks IL-36R agonist binding but also induces broad anti-inflammatory effects on several cells including epithelial cells, DCs, myeloid cells, Th17 cells and regulatory T cells [12, 13].

## Cytokine processing

**Fig. 1:**
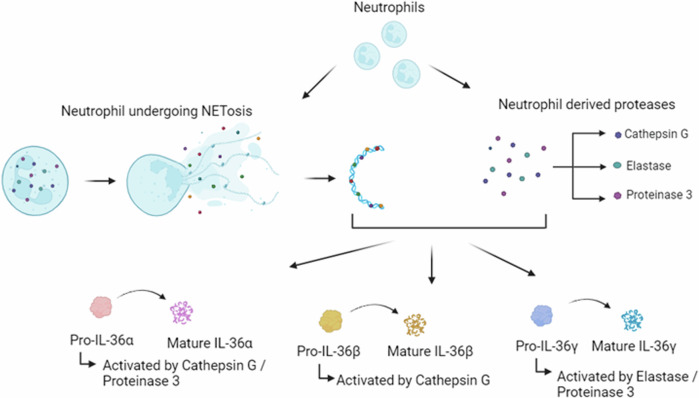
Neutrophil associated IL-36 cytokine processing. Pro-IL-36 cytokines are premature when expressed and require post-translational N-terminal truncation to become biologically active. The neutrophil-derived proteases cathepsin G, proteinase 3 and elastase process pro-IL-36α, pro-IL-36β and pro-IL-36γ. Neutrophil extracellular traps (NETs) are expelled from neutrophils during NETosis. As they contain the same cathepsin G, proteinase 3 and elastase proteases, NETs can directly activate the premature IL-36 cytokines.

Functional activation of the IL-36 family cytokines is carefully regulated due to their significant inflammatory effects. The IL-36 agonists, IL-36RA and IL-38, are expressed as inactive precursors, requiring post-translational proteolytic cleavage to become active. Unlike certain other IL-1 family members, activation of IL-36R ligands is inflammasome independent and instead occurs via N-terminal truncation, which can be achieved by three neutrophil-derived proteases, namely proteinase-3, cathepsin G and elastase (Fig. 1) [14]. IL-36α is activated by cathepsin G or elastase by proteolytic cleavage at lysine 3 and alanine 4. Biological activation of IL-36β is achieved via selective cleavage of arginine 5 by cathepsin G. Finally, IL-36γ is cleaved at valine 15 by elastase [14] and between glutamic acid 17 and serine 18 by cathepsin S [15]. Neutrophil-derived extracellular traps (NETs) provide an additional mechanism for IL-36 activation. NETS are expelled from neutrophils to trap and kill pathogens during infection [16]. They also allow for the release of proteases, which in turn, activate the IL-36R agonists via N-terminal truncation as above (Fig. 1) [17]. IL-36β and IL-36γ also undergo processing and activation in response to diverse allergen-associated proteases, including Aspergillus orzyae and Bacillus Licheniformis, with IL-36α significantly less susceptible to activation by these proteases [18]. N-terminal truncation, as assessed using Biacore binding assay, increased the binding affinity of IL-36α 36,000-fold (K_D_ following truncation = 0.021 nM), increased IL-36β 13,000-fold (K_D_ following truncation = 0.007 nM) and IL-36γ 980-fold (K_D_ following truncation = 0.147 nM) [19]. IL-36RA also requires cleavage to become active. Proteolytic cleavage of the IL-36RA N-terminal methionine by neutrophilic elastase generates a biologically active ligand containing an N-terminal valine residue [19, 20]. Full-length IL-36RA did not bind in the Biacore binding assay, with cleavage resulting in a K_D_ value of 10.2 nM [19].

Similar to the IL-36 cytokines, IL-38 also undergoes N-terminal truncation which induces cytokine maturation [12]. However, unlike other IL-36 family members, the exact proteases involved in proteolytic cleavage of IL-38 have yet to be elucidated.

## Signalling and regulation

**Fig. 2:**
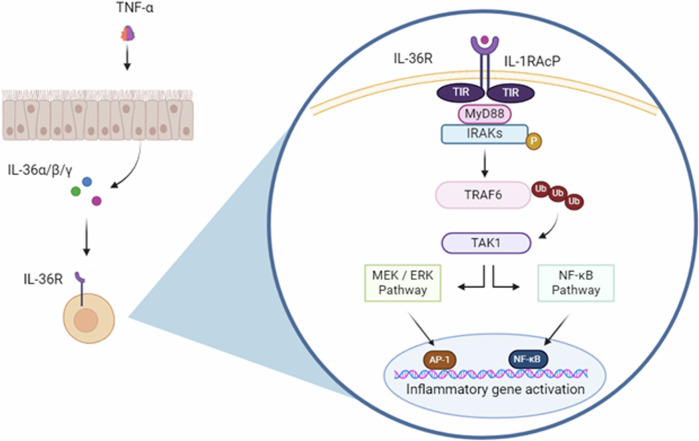
IL-36R signalling following TNF-α stimulated expression of premature IL-36 cytokines. Following processing the mature cytokines bind to the IL-36R – IL-1RacP heterodimeric receptor complex. MyD88 is recruited to this heterotrimeric complex, activating mitogen-activated protein kinase (MAPK) and nuclear transcription factor kappa B (NF-κB) signalling pathways, ultimately regulating inflammatory gene activation.

Following processing, mature IL-36 cytokines bind to the IL-36R [21], inducing the recruitment of the IL-1R accessory protein (IL-1RAcP) and the formation of a heterodimeric receptor complex. Following this, the intracellular toll/interleukin-1 receptor (TIR) binding domains of IL-36R and IL-1RAcP undergo phosphorylation, triggering the recruitment of MyD88 following TIR–TIR interaction. In turn, the IL-1 receptor-associated kinase (IRAK) is recruited, which activates via autophosphorylation, subsequently recruiting the adaptor protein TNF receptor-associated factor 6 (TRAF6) [22]. TRAF6 ligates several ubiquitin molecules, which recruit and activate transforming growth factor-β-kinase 1 (TAK1). These interactions trigger MAPK signalling, leading to activation of the transcription factor AP-1, as well as inducing the phosphorylation of the NF-κB inhibitor, IκB-α, causing the release and activation of NF-κB, which relocates to the nucleus, activating transcription of pro-inflammatory genes [22] (Fig. 2). The IL-36RA inhibits IL-36 cytokine activity by binding to the IL-36R with greater affinity than the pro-inflammatory cytokines, preventing protein dimerization with IL-1RAcP and thus suppressing IL-36R signalling, in a similar manner to the antagonistic activity of IL-1RA [19].

Given the sequence homology IL-38 shares with IL-1RA and IL-36RA, this suggests that IL-38 elicits its antagonistic functions similarly to these receptor antagonists [4]. IL-38 is believed to inhibit the pro-inflammatory activity of the NF-κB and MAPK pathways associated with IL-36R and IL-1R signalling by blocking the binding of their respective agonists, in addition to preventing the recruitment of IL-1RAcP [23]. Recently, another study has suggested that IL-38 can also interact with the orphan receptor, IL-1RAPL1, and inhibit the JNK pathway, thus suppressing inflammation- [12].

## Physiological functions of the IL-36 cytokines

Extensive research has been carried out to determine the physiological functions of IL-36α, IL-36β, and IL-36γ. While the expression of these cytokines in healthy tissue is relatively low, they are highly inducible in the epithelial and epidermal layers of the lungs, skin, brain and gut in response to cellular damage and infection [24–26], pointing towards regulatory and protective functions of these cytokines. The roles of the IL-36R agonists can broadly be described as maintaining barrier homoeostasis through immune regulation and protection against microbial and viral infection.

### Homoeostatic properties

Several studies have shown IL-36 cytokine expression to be involved in maintaining barrier homoeostasis through the activation of wound healing processes. For instance, skin barrier defects in mice resulting from disrupted EGFR signalling have been shown to induce high expression of IL-36α and, to a lesser extent, IL-36β [27]. The resulting increased expression of IL-36α and IL-36β was associated with increased keratinocyte proliferation and immune cell infiltration [27]. Mice engineered to be deficient in expression of the fibroblast growth factor receptor (FGFR) in keratinocytes also demonstrated increased IL-36β production and subsequent keratinocyte hyperproliferation in response to compromised epidermal barriers [28]. IL-36 cytokines also maintain barrier function in additional mucosal tissues, particularly in the intestines and lungs. A study examining IL-36R signalling in a murine model of dextran sodium sulphate (DSS)-induced colitis identified increased expression of IL-36γ and IL-36α, with IL-36R signalling promoting the accumulation and proliferation of fibroblasts [26] and the recruitment of IL-22 producing neutrophil recruitment to the site of the wounds [29]. Further investigation into the role of IL-36R signalling in this context revealed an IL-23/IL-22 cytokine signalling network essential for intestinal barrier repair [30]. Together, these findings highlight the importance of the IL-36 cytokines in maintaining homoeostasis, and how loss of function can lead to broad inflammatory disorders.

IL-36α, IL-36β and IL-36γ also play important regulatory roles in the crosstalk between the innate and adaptive immune system. Epithelial cells, neutrophils and DCs have been identified as a cell triad that is commonly involved in the inflammatory activity of the IL-36 cytokines [31, 32]. All three IL-36 cytokines promote DC maturation, and DCs matured in their presence have an increased ability to promote T-cell proliferation compared to those matured in its absence [32]. They also have been shown to induce Th1 immune responses in naïve T cells [33], along with promoting IFN-I production by plasmacytoid DCs through TLR9 activation [34]. While these functions are important in a healthy host, the dysregulation of IL-36 cytokine signalling is associated with aberrant activation of these and other highly regulated immune mechanisms, leading to the development of severe inflammatory disorders such as generalized pustular psoriasis [35, 36]. It is important to note that this study also found IL-36α and IL-36β to similarly inhibit the differentiation of naïve T cells into T regulatory cells; however, their impact on promoting Th9 cell differentiation was not reported.

### Response to infection

In keeping with their role in upregulating the inflammatory responses, the IL-36 cytokines have been identified as key mediators of the host immune response to infection, through antimicrobial peptide (AMP) production, the promotion of immune cell recruitment and activation, M1 macrophage polarisation, and the promotion of IFN signalling [37–39].

In terms of their role in bacterial infection, IL-36R signalling was shown to be protective in a murine model of polymicrobial sepsis [40] with IL-36R deletion resulting in significantly increased organ injury and mortality, decreased bacterial clearance, and the early apoptosis of lung epithelial cells [40]. IL-36R signalling in macrophages has also been shown to potentially play a role in restricting *Mycobacterium tuberculosis* (*Mtb*) infection in vitro, due to the IL-36γ dependent upregulation of several AMPs including cathelicidin and beta defensin 2 [41] In contrast, an in vivo study using IL-36R-deficient mice failed to demonstrate a role for IL-36 in the defence against *Mtb* infection [42]. IL-36 cytokines are also prevalent in mounting the host response to both fungal and viral infections. Exposure of oral epithelial cells to Candidalysin, a PAMP expressed by *Candida albicans*, resulted in phosphatidylinositol 3 kinase (PI3K) pathway-dependent expression of IL-36γ, followed by downstream production of IL-23 [43]. Mice infected with the influenza virus also experienced significant increases in IL-36γ mRNA levels, with most expression being recorded in neutrophils [44]. The therapeutic benefits of the anti-viral immunity promoted by IL-36 signalling have been realised through the use of truncated IL-36γ as an adjuvant in a Zika DNA vaccine, which resulted in increased protection against Zika challenge through enhanced effector T cell recruitment and function [45]. Of note however, adverse effects of the IL-36 cytokines have also been observed in infection, primarily as a result of overamplification of the inflammatory response [46, 47], highlighting the importance of proper regulation of the IL-36 signalling pathways during all microbial infections.

## Physiological functions of IL-38

Similar to the IL-36 cytokines, IL-38 also regulates the immune response. However, unlike the IL-36R agonists, IL-38 has a tolerogenic role and functions via negative regulation of inflammation. IL-38 primarily functions as an IL-36R antagonist, blocking the binding of IL-36 cytokines and preventing subsequent downstream activation of pro-inflammatory genes [48]. However, it also functions as a cytokine, directly inducing anti-inflammatory effects [12, 13].

Several epithelial, mesenchymal and immune cells upregulate IL-38 expression in response to inflammation, with IL-38 expression found to decrease inflammatory factors and disease pathogenicity in various inflammatory diseases [49–52]. In addition to its expression in response to inflammation, IL-38 is produced by apoptotic cells as a preventative measure to limit inflammation. Truncated IL-38 released by apoptotic cells reduced macrophage production of IL-6 by antagonising the X-linked IL-1R accessory protein-like 1 (IL1RAPL1) along with reducing macrophage-dependent production of IL-17 [12]. Several studies have also identified correlations between polymorphisms in the IL-38 gene, *IL1F10*, and the incidence of inflammatory diseases such as psoriatic arthritis, ankylosing spondylitis, and rheumatoid arthritis [53–55]. In addition to inhibiting the expression of inflammatory factors, IL-38 has been shown to directly promote the production of anti-inflammatory factors including IL-10 and TGF-β1 by Tregs cells, with this effect being enhanced in the presence of LPS [56]. In vitro analysis has also shown IL-38 co-treatment to promote M2 polarisation, reduce apoptosis, and inhibit NLRP3 inflammasome activation of LPS treated macrophages [57].

The protective roles of IL-38 have been examined in multiple disease models. Murine models of DSS-induced colitis showed that administration of rIL-38 to mice with DSS-induced colitis resulted in reduced weight loss and colon damage compared to the control mice along with ameliorated colonic inflammation [58]. The protective role of IL-38 was further shown in an additional murine model of DSS-induced colitis where IL-38 knockout (IL-38KO) mice experienced significantly greater weight loss, shorter colon length and higher histological scores than WT mice [59]. These inhibitory effects on the inflammatory immune response are similarly seen in allergic rhinitis (AR), where IL-38 expression was found to inversely correlate with the expression of IL-17A and IL-23 [13]. Additionally, the regulatory effects of IL-38 on IL-17-mediated inflammation were examined in murine models of imiquimod-induced psoriasis, with IL-38 KO mice displaying increased IL-17 production in addition to increased dermal neutrophil infiltration [60]. In contrast, in a separate study using the same model, IL-38 deficiency did not impact the development or resolution of IMQ-induced skin inflammation [61]. These authors have also recently generated a murine model whereby IL-38 was specifically over-expressed in keratinocytes. They demonstrated that keratinocyte activation and differentiation were not affected by increased IL-38 expression in IMQ-treated skin. There was, however, selective inhibition of CXCL1 and IL-6 production in response to IMQ. These findings indicate that IL-38 may have a complex role in the inflamed skin [62].

These studies highlight the importance of IL-36 and IL-38 in regulating the immune system and inflammation, and how dysregulation of their function can result in the development of several inflammatory disorders. Recently, the roles of both IL-36 and IL-38 in cancer have been the subject of numerous studies, with dual functionality associated with both cytokines.

## IL-36 in cancer

The significance of IL-36 in the pathogenesis of several inflammatory diseases has led to a recent surge in research investigating the pleiotropic roles of these cytokines in cancer. While initial studies pointed towards solely anti-tumorigenic roles, more recent work has identified pro-tumorigenic functions also, suggesting dual functionalities for the IL-36 cytokines in cancer (Table 1).

**Table 1:** An overview of the tumour-suppressing and tumour-promoting roles of the IL-36 family members and IL-38 in cancer.

	Anti-tumorigenic function	Tumour type
**IL-36α**	Suppression of tumour cell proliferation, migration and invasion Enhanced function of tumour infiltrating CD8+ T cells Regulation of NF-κB and MAPK pathways to promote M1 macrophage activity and tumour infiltration Disruption of angiogenesis via HIF-1α downregulation Synergisation with PD-L1 for increased anti-tumour response Expression associated with improved patient prognosis	Human epithelial ovarian [62], human HCC [66], mouse melanoma [63] Human HCC [65], mouse colorectal [69] Mouse melanoma [63] Human NSCLC [72] Melanoma [63] Human epithelial ovarian [62], human thymoma, kidney renal clear cell carcinoma and melanoma [63], human HCC [65, 66], human colorectal [67], human NSCLC [72]
**IL-36β**	Increased tumour infiltration of CD8+ T cells Increased IFN-γ and IL-2 expression by CD8+ T cells via mTORC1 activation Downregulated activity of CD8+ T cell functional inhibitor let-7c-5p Expression associated with improved patient prognosis	Mouse melanoma [64], mouse pancreatic [71] Mouse melanoma [64] Mouse and human pancreatic [71] Human kidney renal clear cell carcinoma and melanoma [63], mouse melanoma [64], mouse pancreatic [71]
**IL-36γ**	Suppression of tumour cell proliferation and migration Increased tumour infiltration of NK and γδ T cells Enhanced function of tumour infiltrating CD8+ T cells Promotion of a type 1 immune response via increased IFN-γ, IL-12 and TNF-α expression Increased sensitivity to CTLA-4 immunotherapy Increased TIL activity Increased expression of PMN-MDSCs Promotion of intra-tumoral TLS formation IL-36γ/IL-23/OX40L mRNA triplet promoted anti-cancer activity and efficiency of checkpoint blockade Expression associated with improved patient prognosis	Mouse melanoma and breast [70], mouse colorectal [80] Mouse melanoma [70], mouse colorectal [80] Mouse breast [70] Mouse melanoma [70] Mouse melanoma [79] Mouse melanoma [73], mouse colorectal [80] Mouse melanoma [73] Mouse colorectal [76] Mouse colorectal [80] Human melanoma and lung [70], human colorectal [97, 98]
**IL-36RA**	Regulation of JNK signalling to inhibit cell adhesion and ECM gene expression Inhibition of Wnt signalling Promotion of CD8+ tumour infiltration Expression associated with improved patient prognosis	Mouse colorectal [85] Mouse colorectal [85] Mouse colorectal [84] Mouse NSCLC [87]
**IL-38**	Regulates ERK pathway to reduce tumour cell proliferation and migration Associated with increased tumour infiltration of CD4+ and CD8+ T cells Associated with reduced PD-1 expression Inhibited inflammatory chemokine production via NF-κB / MAPK disruption Expression associated with improved patient prognosis	Human colorectal [99] Human colorectal [98] Human colorectal [98] Human colorectal [58] Human colorectal [98]
	**Pro-tumorigenic function**	**Tumour type**
**IL-36α**	Promoted inflammatory chemokine expression Promoted tumour cell proliferation Regulated p42/44 MAPK pathway Expression associated with poorer patient prognosis	Human and mouse NSCLC [82] Human and mouse colorectal [84] Human NSCLC [82] Human NSCLC [82]
**IL-36β**	Promoted inflammatory chemokine expression Promoted tumour cell proliferation Regulated p42/44 MAPK pathway	Human and mouse NSCLC [82] Human and mouse colorectal [84] Human NSCLC [82]
**IL-36γ**	Promoted tumour cell proliferation Regulated p42/44 MAPK and P13K/AKT pathways Synergised with the IL-17A/IL-22 signalling axis Promoted inflammatory chemokines expression (CCL2, CCL20, CXCL1, LCN2, IL-8) Regulated JNK signalling to promote cell adhesion and ECM gene expression Regulated Wnt signalling Neutralised reactive oxygenase species Promoted epithelial cell transformation Promoted tumoral expression of PD-L1 Expression associated with poorer patient prognosis	Human and mouse colorectal [84, 85], human NSCLC [82], human gastric [83], human oral squamous cell carcinoma [86] Human colorectal [84], human NSCLC [82] Human colorectal [81], human NSCLC [82] Human and mouse NSCLC [82], human colorectal [85] Human and mouse colorectal [85] Mouse colorectal [85] Mouse NSCLC [87] Human breast [88] Human NSCLC [82] Mouse NSCLC [87], human colorectal [67], human NSCLC [82], human gastric [83]
**IL-36RA**	Associated with increased intra-tumoral PD-1, PD-L1 and CTLA4 expression	Human colorectal [77]
**IL-38**	Regulated JNK/AP-1 signalling to promote cell proliferation, migration and inflammatory chemokine and cytokine production Reduced tumour infiltration of CD8+ T cells Reduced tumour infiltration of NK and B-cells Expression associated with poorer patient outcome	Human and mouse squamous cell carcinoma [96] Human prostate [92], mouse lung [94], mouse breast [95] Squamous cell carcinoma [93] Human breast cancer [95], human NSCLC [90], human prostate [92]

### IL-36 as a tumour suppressor

Increased IL-36 cytokine expression levels, in particular IL-36α, have been correlated with improved prognosis in several cancers including ovarian cancer [63], melanoma [64, 65], hepatocellular carcinoma (HCC) [66, 67], and colon adenocarcinoma [68]. IL-36α expression was downregulated in epithelial ovarian cancer (EOC) tumour tissue compared to adjacent healthy tissue, with further analysis revealing an inverse correlation between IL-36α expression and EOC cell proliferation, migration and invasion. Overexpression of IL-36α resulted in the suppression of these pro-tumorigenic phenotypes in EOC cells, while inhibition of IL-36α via small interfering RNA (siRNA) promoted tumorigenesis [63]. In HCC, decreased expression of IL-36α was associated with greater tumour size and poorer patient prognosis, while increased expression was associated with a greater number of tumour infiltrating CD3^+^CD8^+^ T cells [66]. In a more recent study examining IL-36 in HCC, IL-36α expression was increased in cancer tissue relative to normal tissue, along with being positively correlated with cancer differentiation state. In addition, in vitro analysis revealed that exogenous IL-36α inhibited HCC viability in a dose-dependent manner, while also having an inhibitory effect on cell migration [67].

The relationship between the expression of IL-36β and IL-36γ in cancer and patient outcome has not been as extensively reported as IL-36α, instead the functional anti-tumorigenic roles of the IL-36 cytokines, particularly IL-36γ, have been more extensively reported. IL-36 cytokine signalling has displayed distinct protective functions via the promotion of anti-tumorigenic inflammatory immune responses. [63–65, 69] In a murine model of colorectal cancer (CRC), subcutaneous injection of IL-36α overexpressing CT26 cells resulted in a significant reduction in tumour weight, volume, decreased lung metastasis, and increased infiltration of CD3^+^CD8^+^ TILs compared to the control CT26 cell tumours [70]. Anti-tumorigenic roles of IL-36α have also been reported in melanoma, including the promotion of pro-inflammatory M1 macrophage activation and tumour infiltration [64]. Tumours containing IL-36α overexpressing B16 cells also displayed increased infiltration of MHC II^high^ macrophages compared to control B16 tumours. Furthermore, this study found that the combination of PD-L1 antibody treatment with IL-36α resulted in greater inhibition of tumour growth than treatment with either anti-PD-L1 or IL-36α on their own [64].

Similar to IL-36α, IL-36γ has been shown to promote immune-mediated anti-tumorigenic activity in several cancers. A B16-IL-36γ overexpressing murine model of melanoma showed inhibited tumour growth and increased survival compared to mice injected with wildtype B16 cells. These tumours displayed increased infiltration of natural killer (NK) and γδ T cells, in addition to increased expression of various cytokines consistent with a type 1 immune response including IFN-γ, IL-12 and TNF-α [71]. IL-36γ expression also suppressed the growth and metastasis of 4T1-IL-36γ murine breast cancer cells in vivo [71].

The mammalian target of rapamycin complex 1 (mTORC1) has been identified as a central mediator of the anti-tumorigenic effects of IL-36β [65]. B16 melanoma cells overexpressing IL-36β resulted in increased CD8^+^ TIL activity. This was mediated via activation of mTORC1 and resulted in the expression of IFN-γ and IL-2 by CD8^+^ T-cells [65]. More recently, microarray analysis of IL-36β in pancreatic cancer has revealed that intra-tumoral injection of IL-36β results in downregulation of let-7c-5p, a microRNA responsible for the downregulation of IL-2 and IFN-γ in CD8^+^ T-cells [72], increasing understanding of the underlying mechanism by which IL-36β activates CD8^+^ T cells.

The IL-36 cytokines have also displayed anti-tumorigenic functions through the disruption of tumour-promoting mechanisms. In non-small cell lung cancer (NSCLC), IL-36α appeared to disrupt angiogenesis via downregulation of HIF-1α, a transcription factor of VEGFA, a major inducer of angiogenesis [73]. Treatment of cells overexpressing IL-36α with VEGFA reversed this inhibition, confirming that the angiogenic action of IL-36α is through VEGFA dysregulation [73]. In addition to the potent effects of the IL-36 cytokines on the adaptive immune response, research shows IL-36R signalling to significantly enhance the anti-tumorigenic innate immune response. Intra-tumoral administration of recombinant IL-36γ (rIL-36γ) in a murine model of an immunologically cold melanoma tumour resulted in increased conventional type 1 and type 2 DCs (cDC1, cDC2), F4/80^+^ macrophages, Ly6C^+^ monocytes and neutrophils compared to control B16F10 tumours [74]. IL-36R signalling on neutrophils was specifically highlighted as promoting broad anti-tumorigenic effects, namely decreasing expression of polymorphonuclear myeloid-derived suppressor cells (PMN-MDSC) suppressing genes, increased neutrophil migration to the TME, promoting T-cell chemoattractant CXCL10 expression, in addition to increasing the cytotoxicity of neutrophils by enhancing the expression of reactive oxygen species (ROS) and granzyme B [74].

IL-36γ has been proposed to further regulate the anti-tumour immune response via tertiary lymphoid structure (TLS) formation, which are lymphoid organs composed of innate and adaptive immune cells which develop ectopically in inflamed tissues and can be associated with positive prognosis in cancer [75, 76]. The involvement of IL-36γ in optimum TLS anti-tumorigenic activity was originally identified in a murine model of colon carcinoma following intra-tumoral injections of DCs engineered to overexpress the IL-36 cytokine [77]. DC.IL-36γ injected WT mice displayed increased intra-tumoral TLS development along with decreased tumour growth, while the therapy was ineffective in IL-36R^−/−^ mice [77]. The involvement of IL-36γ in TLS formation has also been proposed in human CRC. Immunohistochemistry analysis on primary human CRC tumours revealed a correlation between high expression of IL-36γ by vascular endothelial cells (VECs) in the vasculature surrounding the TLS and increased TLS composition of CD20^+^ B-cells [78]. However, the TLSs examined were all located in the invasive margin (IM), the area surrounding the tumour separating healthy (peri-tumoral) tissue and malignant tissue. Indeed, a large-scale analysis on the prognostic value of TLSs in over 600 patients with CRC liver metastasis revealed no correlation between TLS expression in the IM and relapse-free survival (RPS)/overall survival (OS) [79]. In contrast, significant correlations were observed between high numbers of intra-tumoral TLSs and increased RPS/OS [79]. It is, therefore, necessary to further examine the association between IL-36γ and TLS in the context of tumour suppression.

In terms of additional potential therapeutic benefits, recent work suggests that the IL-36 cytokines may enhance the efficacy of checkpoint inhibitor immunotherapy. Murine models of melanoma generated using IL-36γ overexpressing B16 cells displayed increased IFN-γ production and proliferation of CD8^+^ and CD4^+^ T cells following treatment with CTLA-4 monoclonal antibodies, compared to CTLA-4 or IL-36γ treatment alone [80]. Moreover, in a murine study in CRC exploring the suitability of direct intra-tumoral delivery of IL-36γ, IL-23 and the T-cell costimulatory ligand OX40 (OX40L) mRNAs [81] showed activation and proliferation of various immune cell types including natural killer T (NKT) cells, δγ T cells, CD4+, and CD8+ T cells, in addition to inhibiting metastasis, promoting TIL activity, and improving the efficacy of checkpoint blockade therapy [81]. These are promising findings as TILs are functionally suppressed by the immune checkpoint activity of Treg cells.

### IL-36 as a tumour promotor

In contrast to the broad anti-tumorigenic roles reported for the IL-36 cytokines, predominantly associated with their ability of these cytokines to recruit type 1 immune populations to the TME, recent studies have reported distinct tumour-promoting roles in various cancers, highlighting the pleiotropic nature of these cytokines. An examination of the of IL-36 cytokines in CRC revealed that distinct expression levels were associated with specific patient prognosis, with high IL-36α expression and low IL-36γ expression both found to be associated with better patient outcome [68], possibly indicating divergent roles for these cytokines in CRC. More recently, transcriptomic analysis of the IL-36 cytokines in CRC demonstrated that decreased tumoral expression of the IL-36R was associated with improved patient outcomes [82]. Of note this study also identified that the most consistent and significant increases in IL-36R expression occur in epithelial-originating cancers, including lung squamous cell carcinoma, colon, oesophageal and stomach adenocarcinomas, indicating a possible correlation between IL-36R expression and patient outcomes in these cancers. In contrast, invasive breast carcinoma, prostate adenocarcinoma, and glioblastoma showed consistently lower levels of IL-36R expression [82]. Furthermore, analysis of lung adeno- and squamous cell carcinomas revealed increased protein expression of all three IL-36 agonists and the IL-36R relative to normal tissue. IL-36α and IL-36γ protein expression were localised to the epithelium, with increasing expression correlating with increasing tumour grade, while IL-36β protein expression was found in the immune compartment and was only present in normal and higher-grade tumours [83]. Similarly, in stomach adenocarcinoma, a correlation has been identified between increased IL-36γ expression and worse patient outcomes. This study also showed that increased IL-36RA expression correlated with worse patient outcomes [84]. Unlike in CRC, IL-36γ expression was not found to correlate with tumour stage in stomach adenocarcinoma [84].

In terms of the cancer promoting activities of the IL-36 cytokines, several studies have now demonstrated their involvement in promoting pro-tumorigenic cell proliferation, migration, invasion, chemokine expression and in supporting an immune suppressive TME. In vitro analysis has shown that IL-36γ promotes migration and invasion in AGS (primary gastric adenocarcinoma), MKN1 (lymph node), and MKN45 (liver metastasis) cells [84]. IL-36γ has also been shown to promote proliferation in HT29 and CT26 (human and murine CRC) [85], HCT116 and MC38 (human and murine CRC) [86], LLC (human NSCLC) [83], and HSC-3 (human oral squamous cell carcinoma) [87] cells. IL-36-induced proliferation was shown to be mediated via the ERK signalling pathway in several of these studies [84, 85]. The IL-36R agonists additionally elicit pro-tumorigenic effects through activating the expression of inflammatory chemokines. Increased expression of CCL2 and CCL20 was found in LLC and SKMES-1 NSCLC cells, respectively following stimulation with IL-36α, IL-36β, or IL-36γ [83]. Moreover, IL-36γ and IL-17A were found to synergistically induce expression of CXCL1 and CCL20 in HT29 cells, with an additive effect on proliferation. IL-36γ and IL-22 were also found to synergistically induce expression of CXCL1 and LCN2 [82]. This synergism between IL-36γ and IL-17A/IL-22 was found to also be present in NSCLC, along with a strong synergistic effect between IL-36 and TNFα in the production of CXCL1, IL-8 and CCL20 [83].

Several studies have also shown that the IL-36 cytokines can elicit pro-tumorigenic effects in vivo. A study examining tumorigenesis in several murine models of CRC (AOM/DSS model, AOM/Villin-Cre;*Trp*53^fl/fl^ and APC^Min/+^ model) reported distinct tumour-promoting and tumour-suppressive functions of IL-36γ (encoded by *Il1f9*) and IL-36RA (encoded by *Il1f5*), respectively [86]. Phenotypically, *Il1f9*^−/−^ mice displayed reduced weight loss, fewer and smaller tumours, along with increased recovery rates compared to *Il1f9*^+/+^ mice. In contrast, *Il1f5* knockdown yielded increased weight loss and larger tumours with lower survival rates compared with *Il1f5*^+/+^ mice. IL-36γ stimulation of WT mouse organoids induced upregulated expression of cell adhesion and extracellular matrix-associated genes including *Col6a1, Col4a1, Col1a1* and *Vwf* in mice in a JNK-dependent manner, while IL-36Ra stimulation downregulated their expression. The expression of IL-36γ and Col6a1 were similarly found to be correlated in human CRC biopsies [86]. IL-36γ and IL-36RA were further found to regulate Wnt signalling in DSS and AOM/DSS treated mice by inducing and impairing expression of Wnt genes *Dab2, Cd44, Tcf7* and *Wnt9a*, respectively [86]. These results suggest that IL-36γ signalling promotes a pro-tumorigenic environment via disruption of the TME. Consistent with this, two additional models of CRC, one using subcutaneous injection of CT26 cells followed by IP injection of recombinant IL-36Ra and the other using subcutaneous injection of IL-36R knockout CT26 cells, both displayed reduced tumour burden and increased tumour infiltration of CD8^+^ T cells [85]. Similarly, the induction of a murine model of NSCLC in IL-36γ knockout mice resulted in a significant attenuation in tumour progression along with increased survival [88]. This study reported a novel TME altering mechanism involving IL-36γ-dependent activation of genes involved in glutathione metabolism, which counteract oxidative stress and related cell death by neutralising reactive oxidative species (ROS). The dependency of this mechanism on IL-36γ signalling was demonstrated by its inhibition by the administration of z-Ala-Pro-Ile (API), a peptide which blocks the N-terminal cleavage essential for IL-36 cytokine maturation and activity. Inhibition of IL-36γ subsequently increased survival and inhibited tumour progression [88].

IL-36γ signalling also promotes epithelial cell transformation in breast tumorigenesis via phosphorylation of JNK1/2, MEK1/2 and ERK1/2, resulting in the subsequent activation of c-Jun, c-Fos and AP-1 transcription factors [89]. This same study also identified that the pro-tumorigenic effects of IL-36γ were enhanced by the activity of peptidyl-prolyl cis-trans isomerase NIMA-interacting 1 (PIN1), while PIN1 knockout significantly attenuated IL-36γ activity [89]. Baker et al., also identified a novel pro-tumorigenic role of IL-36γ via inducing expression of the immune checkpoint inhibitor PD-L1 on SKMES-1 cells, directly aiding in the development of an immune suppressive environment [83]. This contrasts the findings reporting a direct correlation between increased IL-36RA and PD-L1 expression in CRC tissue [78].

**Fig. 3:**
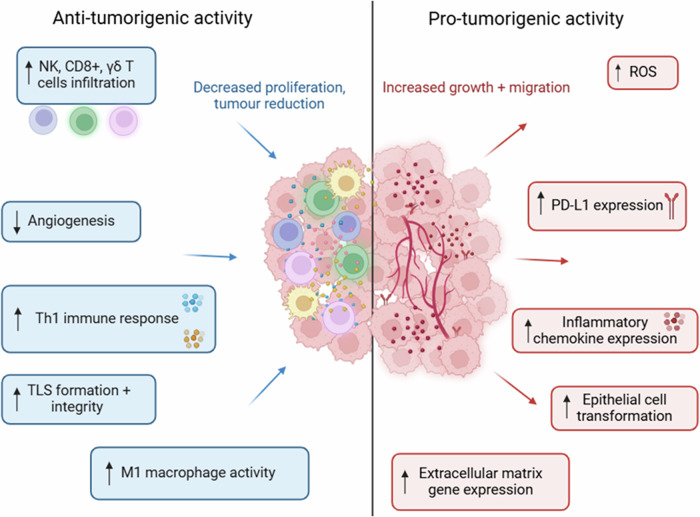
An overview of the potential anti- and pro-tumorigenic activity of the IL-36 and IL-38 cytokines. These cytokines (particularly IL-36γ) display broad anti- and pro-tumorigenic roles across a variety of malignancies. The anti-tumorigenic functions involve enhancing the suppressive activity of the immune system. In contrast, the pro-tumorigenic functions promote immune evasion and cell proliferation.

The IL-36 cytokines display broad anti- and pro-tumorigenic functions in cancer (Fig. 3). Based on these reported findings, IL-36α, IL-36β and IL-36γ present promising therapeutic targets in CRC and NSCLC, although any therapeutic development must be considered in the context of these cytokines’ ability to also act as tumour suppressors. It is important to note that these contrasting roles in cancer are not limited to the IL-36 family of cytokines, with several members of the IL-1 superfamily displaying similar functional variations [90].

## IL-38 in cancer

While the role of IL-38 in cancer has not been as extensively studied as IL-36, the available literature shows similarly dichotomous pro- and anti-tumorigenic functions for IL-38. In contrast to IL-36 cytokines, which have both pro- and anti-tumorigenic effects in the same cancer, the role of IL-38 appears, thus far, to be cancer-type-dependent.

### IL-38 as a tumour promotor

Broad pro-tumorigenic functions of IL-38 have been observed in several cancers, particularly through suppression of the anti-tumorigenic immune response. In primary lung adenocarcinoma, high IL-38 expression was found to be significantly associated with TNM stage and PD-L1 expression. High IL-38 expression was also associated with shorter DFS and OS post-surgery [91]. Increased IL-38 was also observed in the serum of patients with both low- and high-grade primary brain tumours relative to healthy individuals [92]. Similarly, in prostate cancer high IL-38 expression correlated with increasing TNM stage, with multivariate analysis indicating that IL-38 expression could be a reliable biomarker for predicting patient survival [93]. An inverse correlation was identified between IL-38 and CD8+ T cell infiltration, indicating that the pro-tumorigenic functions of IL-38 may involve suppression of the immune response [93]. This downregulation of tumour-infiltrating immune cells by IL-38 was also shown in squamous cell carcinoma, where an inverse correlation between tumoral IL-38 expression and immune cell infiltration of NK, T-cells and B-cells was identified [94]. A study examining the function of IL-38 in lung cancer found that mice bearing IL-38 transfected LLC cells experienced significantly increased tumour volumes compared to mice with control cells [95]. Further analysis revealed a significant decrease in the number of CD3^+^ and CD8^+^ TILs in IL-38-expressing LLC cells, in addition to significantly decreasing the expression of TNFα, IL-17A and IFN-γ [95]. A recent study using the polyoma virus middle T oncoprotein (PyMT) murine model of breast cancer found that IL-38 inhibition resulted in reduced tumour burden along with an increase in the activation and tumour infiltration of CD8+ T cells and γδ T cells. Blocking of the γδ TCR in IL-38 KO mice disrupted the cDC1-dependent recruitment of tumour-infiltrating CD8+ T cells, suggesting IL-38 signalling inhibits these interactions [96]. Furthermore, this study highlighted the potential of IL-38 inhibition as a co-treatment with chemotherapy, with combined treatment resulting in smaller tumour sizes and increased TIL infiltration compared to chemotherapy alone [96].

In contrast to these cancers whereby IL-38 acts via suppression of the anti-tumour immune response, in a murine model of DMBA/TPA-induced squamous cell carcinomas (cSCC), IL-38 was shown to induce expression of inflammatory cytokines and drive proliferation and migration of cancer cells in an IL-36R-dependent manner. Knockout of IL-38 in keratinocytes resulted in a reduced number and volume of tumours, along with reduced recruitment of several immune cells to the site of tumorigenesis, particularly macrophages and CD4+ T cells. In addition, the expression of several pro-inflammatory cytokines and chemokines such as IL-1β, IL-22, TNF-α, CXCL1, CXCL2, CCL17 and CCL20 were reduced in IL-38KO mice [97], suggesting that IL-38 promotes tumour growth and development in cSSC through promoting an inflammatory tumour environment. In line with these findings, these authors found downregulation of Il-38 expression in human cSSC [97].

### IL-38 as a tumour suppressor

For many years, IL-38 was believed to act solely as a tumour promotor. However, anti-tumorigenic functions of IL-38 have recently been observed in CRC. In a recent study, IL-38 expression was reported to be consistently downregulated by approximately 95% in CRC tissue compared to adjacent normal tissue, with higher IL-38 expression correlating with better survival rates [98]. This study also identified the potential for IL-38 as a biomarker for poorly differentiated CRC [98]. Another study examining CRC found high IL-38 expression in colorectal regional nodes to be associated with greater overall survival, increased CD4^+^ and CD8^+^ T expression and reduced PD-1 expression [99]. In addition, inverse correlations were identified between IL-38 expression and TNM stage/tumour differentiation [99]. IL-38 also effectively inhibits IL-36 cytokine-induced expression of inflammatory chemokines including CXCL1, CXCL2 and CXCL9 in HT29 and T84 CRC cells in a dose-dependent manner via disruption of IL-36R NF-κB/MAPK signalling [59]. IL-38 also disrupted ERK signalling in LoVo cells (a grade four CRC cell line), leading to reduced cellular proliferation and migration [100]. Transgenic overexpression of IL-38 in AOM/DSS murine models of CRC resulted in reduced expression of Ki-67 and P-ERK, confirming that IL-38 suppresses CRC via disruption of the ERK signalling pathway and cell proliferation [100].

Collectively these findings show that, similar to its IL-36 cytokine counterparts, IL-38 displays distinct pro- and anti-tumorigenic functions in cancer. Recent years have seen a significant increase in the interest surrounding the role of IL-38 in cancer, with the involvement of IL-38 in several of these malignancies potentially highlighting the suitability of this cytokine as a cancer diagnostic or progression biomarker.

## Conclusions and future perspectives

IL-36α, IL-36β, IL-36γ and IL-38 are multifaceted cytokines, displaying both pro- and anti-tumorigenic functions, sometimes even in the same cancer type. Whilst these cytokines are structurally conserved, this review has highlighted certain unique features of the agonistic IL-36 cytokines in terms of their roles in either cancer development or tumour suppression. IL-36α has generally been shown to induce the mildest pro-tumorigenic effects, seeming to have a more dominant role in the anti-tumorigenic immune response. IL-36γ is the cytokine most reported upon to have pro-tumorigenic effects but this is possibly not surprising as it is, to date, the most studied of the agonists in terms of its pro-tumorigenic phenotype. Interestingly, however, in the studies that investigated all three agonists in a side-by-side manner, IL-36γ consistently showed the strongest activity in terms of its ability to drive cellular migration, proliferation and invasion in vitro as well as inducing higher levels of chemokines associated with tumour progression [82, 83, 86]. Given that the three IL-36 agonists share the same signalling pathway and indeed that the activity (EC_50_) of IL-36γ is reported to be lower than that of IL-36α and IL-36β [19], further work is required to better understand why IL-36γ appears to exert the stronger pro-tumorigenic function and to determine whether this translates to the in vivo situation.

This review highlights that certain models clearly demonstrate the ability of IL-36 cytokines to exert pro-tumorigenic effects whilst utilisation of other models allows for the anti-tumorigenic effects to dominate. Many of the publications that have reported on the anti-tumorigenic effects of the IL-36 cytokines have utilised murine models in which tumour cells have been engineered to overexpress high levels of these cytokines, thereby facilitating recruitment of immune populations important in the anti-tumour immune response. Similarly, other murine models, reported by us and others examining the anti-tumorigenic effects of IL-36 cytokines, utilised intraperitoneal injection of the cytokines or knockout models. As none of these models fully recapitulates the true in vivo situation, the relative contribution of each of these cytokines to either drive tumour progression or facilitate the anti-tumour immune response, particularly in human cancers and in the different cancer types, remains to be fully determined.

An additional question concerning the role of IL-36 in cancer is whether IL-36 cytokines effect their pro-or anti-tumorigenic phenotypes at different stages of tumour development. As shown in Table 1, conflicting findings have been identified for these cytokines within the same tumour types, possibly reflecting that at certain stages of tumour development L-36 may exert a pro-tumorigenic effect, whilst at other stages of development its anti-tumorigenic properties might dominate. An additional possibility is that the pro-tumorigenic effects might prevail in some cancer types, whereas in others, the anti-cancer phenotype might be more evident Indeed, whilst the anti-tumorigenic effects of IL-36 have been reported in multiple tumour types, to date, the pro-tumorigenic effects of IL-36 have been predominantly reported in cancers from epithelial cells associated with mucosal surfaces such as GI, lung and oral cancers. It is clear that more studies are needed utilising human samples and human cancer models to better understand the pro- versus anti- tumorigenic functions of these cytokines. It is possible that spatial immunophenotyping of IL-36 in human cancers could aid in the better understanding of the true role of these cytokines in vivo. Such studies would allow for an in-depth examination of where in the tumour microenvironment these cytokines are expressed, the intensity of expression, and whether expression correlates with tumour grade and stage and response to treatment. Such studies may help to unravel the overarching questions associated with identification of the factors that determine the role of IL-36 in cancer.

With respect to the interplay between IL-36 and IL-38 in different cancer types and the role that IL-38 plays in inhibiting the action of IL-36 in cancer, studies highlighted in this review demonstrate that, in certain cancers, there does indeed seem to be a relationship between these two cytokines in terms of their effect on tumour development/tumour suppression. For example, IL-36 has been shown to have anti-tumour effects on breast cancer development due to enhanced CD8^+^ TIL activity [71], and correspondingly, IL-38 has been reported to have pro-tumorigenic effects in breast cancer due to a reduction in CD8^+^ TIL activity [96]. Both cytokines also respectively suppress and drive tumour growth in lung cancer [73, 95]. However, highlighting the complexity of these cytokines, correlations can also be identified in the opposite direction, with the ability of IL-36 to promote its pro-tumorigenic effects in colorectal cancer (proliferation, migration) shown to occur through activation of the, p42/44 MAPK and P13K/AKT pathways [85] with IL-38 reported to regulate the ERK pathway in colorectal cancer in order to reduce tumour cell proliferation and induce an antitumour effect [100]. These examples show that the effects of the IL-36 cytokines in cancer seem firmly to lie with the ability to either recruit immune cells to the tumour to drive anti-tumour effects, with IL-38 limiting this recruitment. Alternatively, IL-36 drives a pro-tumorigenic effect through its interaction directly on the cancer cells, with IL-38 inhibiting this pro-tumorigenic effect [59]. As above, only by determining which of these scenarios dominates in human cancer types will the role of these cytokines in cancer be clearly identified. The above findings, particularly more recent work, suggest the potential for the IL-36 cytokines as therapeutic targets in IL-36R-expressing cancers. Current research indicates that IL-38 also shows promise as a diagnostic biomarker and a treatment for CRC, while showing potential as a target in lung adenocarcinoma. Ultimately, however, further research on IL-38 in various other cancers is necessary to examine if its observed pattern of promoting discrete pro- and anti-tumorigenic phenotypes in cancer holds up or if, like its IL-36 cytokine counterparts, it can induce both phenotypes in the same cancer. However, while significant research has been done on the mechanisms employed by these cytokines in driving either a pro-or anti- tumorigenic phenotype, the specific factors that determine the role these cytokines will play in cancer, how their role is influenced by the TME and the conditions under which their pro- or anti-tumorigenic effects predominate, have yet to be elucidated. A better understanding of such factors is necessary to allow for the development of these cytokines as cancer therapies.
